# One-step versus two-step screening for diagnosis of gestational diabetes mellitus in Iranian population: A randomized community trial

**DOI:** 10.3389/fendo.2022.1039643

**Published:** 2023-02-02

**Authors:** Fahimeh Ramezani Tehrani, Maryam Rahmati, Farshad Farzadfar, Mehrandokht Abedini, Maryam Farahmand, Farhad Hosseinpanah, Farzad Hadaegh, Farahnaz Torkestani, Majid Valizadeh, Fereidoun Azizi, Samira Behboudi-Gandevani

**Affiliations:** ^1^Reproductive Endocrinology Research Center, Research Institute for Endocrine Sciences, Shahid Beheshti University of Medical Sciences, Tehran, Iran; ^2^Non-Communicable Diseases Research Center, Endocrinology and Metabolism Population Sciences Institute, Tehran University of Medical Sciences, Tehran, Iran; ^3^Infertility and Cell Therapy Office, Transplant & Disease Treatment Center, Ministry of Health and Medical Education, Tehran, Iran; ^4^Obesity Research Center, Research Institute for Endocrine Sciences, Shahid Beheshti University of Medical Sciences, Tehran, Iran; ^5^Prevention of Metabolic Disorders Research Center, Research Institute for Endocrine Sciences, Shahid Beheshti University of Medical Sciences, Tehran, Iran; ^6^Faculty of Medicine, Shahed University, Tehran, Iran; ^7^Endocrine Research Center, Research Institute for Endocrine Sciences, Shahid Beheshti University of Medical Sciences, Tehran, Iran; ^8^Faculty of Nursing and Health Sciences, Nord University, Bodø, Norway

**Keywords:** gestational diabetes, maternal and neonatal outcomes, one-step screening approach, prevalence, two-step screening approach

## Abstract

**Objectives:**

There is considerable worldwide controversy regarding optimal screening and diagnostic approaches for GDM. This study aimed to compare the prevalence, maternal and neonatal outcomes of a One-step with a Two-step approach for the screening and diagnosis of GDM in a large community sample of pregnant women.

**Methods:**

We conducted a secondary analysis of a randomized community non-inferiority trial of GDM screening in Iran. For the current study, all pregnant women who met the inclusion criteria were randomized into two groups for GDM screening. The first group of women (n = 14611) was screened by a One-step screening approach [75-g 2-h oral glucose tolerance test (OGTT)] and the second group (n = 14160) by a Two-step method (the 50-g glucose challenge test followed by the 100-g OGTT). All study participants were followed up until delivery, and the adverse maternal and neonatal outcomes were recorded in detail.

**Results:**

GDM was diagnosed in 9.3% of the pregnant women who were assigned to the One-step and in 5.4% of those assigned to the Two-step approach with a statistically significant difference between them (p < 0.001). Intention-to-treat analyses showed no significant differences between the One-step and the Two-step group in the unadjusted risks of the adverse pregnancy outcomes of macrosomia, primary cesarean-section, preterm birth, hypoglycemia, hypocalcemia, hyperbilirubinemia, preeclampsia, neonatal intensive care unit admission, birth trauma, low birth weight, and intrauterine fetal death. Results remained unchanged after adjustment for potential confounder variables including gestational age at enrollment and delivery, maternal body mass index, gestational weight gain, type of delivery, treatment modality, and GDM diagnosis in the first trimester.

**Conclusion:**

We found that although the rates of GDM more than doubled with the One-step strategy, the One-step approach was similar to the Two-step approach in terms of maternal and neonatal outcomes. These findings may warn that more caution should be exercised in adopting the One-step method worldwide. Future research is needed to assess the long-term harm and benefits of those approaches to GDM screening for both mothers and their offspring.

**Clinical trial registration:**

https://www.irct.ir/trial/518, identifier (IRCT138707081281N1).

## Introduction

Gestational diabetes mellitus (GDM) is one of the common morbidities in pregnancy (1), with short and long-term maternal, fetal, and newborn adverse outcomes (2–5). The pooled global standardized prevalence of GDM was 14.0% ranged between 7.1% and 27.6% (6).

Although there is no doubt about the effectiveness of GDM screening and treatment in reducing the risk of adverse outcomes (7–10), there is considerable worldwide controversy regarding optimal screening and diagnostic approaches for GDM. In this respect, major scientific bodies had different recommendations. The American College of Obstetricians and Gynecologists (ACOG) endorsed a Two-step approach, starting with an initial 50-g glucose 1-h challenge test (GCT), followed, if the GCT is abnormal, by a 100-g oral glucose tolerance test (OGTT) (11). Besides, the International Association of Diabetes in Pregnancy Study Group (IADPSG) recommends a One-step approach, based on 75-g, 2-h oral glucose tolerance test, to diagnose GDM (12). As well, although firstly the World Health Association (WHO) adopted One-step GDM screening, based on 75-g, 2-h oral glucose tolerance test at second trimester of gestation (13), it currently does not have a recommendation on whether or how to screen for GDM (14); however, all of them highlighted the need for additional evidence for more confirmation.

However, despite several recent large-scale studies, debate continues addressing which of these two clinically recommended screening approaches may better improve pregnancy outcomes (1, 15–22). Recently, two meta-analyses comparing the One-step and Two-step approaches in terms of adverse pregnancy outcomes were published, which had conflicting results (18, 21). Saccone et al. (18) in a meta-analysis of four randomized clinical trials (RCTs) (n = 2582 participants), reported that diagnosis of GDM by the One-step approach was associated with better perinatal outcomes, compared to the Two-step approach (18). In contrast, Brady et al. (21) in a meta-analyses of four RCTs (n = 24,966 patients) and 13 observational studies (n = 710,677 patients), found that despite a significant increase in GDM diagnosis and treatment with One-step testing, there is no difference in rate of LGA neonates compared with Two-step testing among RCTs. Additionally, in the analysis of high-quality RCTs and observational studies, One-step testing was associated with a lower rate of LGA neonates (pooled RR 0.97; 95% CI 0.95–0.98), but higher rates of GDM diagnosis, treatment, NICU admission, and neonatal hypoglycemia (21).

The National Institutes of Health GDM consensus conference proposed a large randomized trial to compare these two approaches with respect to important adverse pregnancy outcomes in different population (23). Furthermore WHO strongly recommended that screening strategies for GDM should be considered a priority area for research, particularly in in low- and middle-income countries such Iran (14).

Therefore this study aimed to compare the prevalence and maternal and neonatal outcomes of a One-step with a Two-step approach for the screening and diagnosis of GDM in a large community sample of Iranian pregnant women.

## Materials and methods

We conducted a secondary analysis of a randomized, non-inferiority trial among Iranian pregnant women. The protocol was approved by the national ethics committee of the National Institute for Medical Research Development (Approval number: IR.NIMAD.REC.1394.013). In addition, the Iranian Ministry of Health and Medical Education (MoHME) approved the study protocol, and pre-specified GDM modalities were made available to all those provinces as mandatory guidelines. Detailed methods have been described elsewhere (24, 25). Briefly, a total of 35,613 pregnant women in the first trimester of pregnancy from five different geographic regions of Iran were recruited. The pregnant women with the following criteria were excluded: maternal age less than 18 years, history of preexisting diabetes or other chronic disorders, and uncertainty about the date of last menstrual period among those who did not have first trimester ultrasound estimation.

Cluster randomization was stratified by five geographic regions (North, East, West, South, and Center) of Iran. One province in each stratum was randomly selected (Golestan, South Khorasan, Kurdistan, Bushehr, and Yazd, respectively); in the next step, all cities in each province were classified in two clusters of the center of the province and other cities and after that four cities were randomly selected from the list of other cities in each province. Finally, five different protocols were randomly allocated to each provincial center. Also, in the cluster of other cities, four other cities in each province were randomly allocated to the rest of the protocols (**Supplementary Table 1**) (24, 25). Along with routine prenatal standard care, all participants underwent two phases of GDM screening in the first and second trimester of pregnancy, based on fasting plasma glucose (FPS) levels in the first and either a One-step or a Two-step screening method in second trimester of pregnancy. The value of at the FBS for GDM detection in the first trimester was based on ISDPSG-2016 recommendation at the time of study design.

For the current study, all pregnant women who classified as non-GDM in the first trimester of pregnancy were randomized for GDM re-screening between 24 and 28 weeks, using either a One-step (n = 14611) or Two-step (n = 14160) approach. In this respect, One-step screening was based on a 75 gram 2-h oral glucose tolerance test (75 g 2h-OGTT). Participants were labeled as GDM if at least one values exceeded the cut-off, including fasting plasma glucose ≥ 92mg/dl, but <126 mg/dl and/or 2-h OGTT ≥153 mg/dl. The Two-step approach was as follows: firstly, a 50 g oral glucose challenge test (GCT) was performed regardless of the fasting status. One-hour plasma glucose level ≤140 mg/dl was considered negative and needed no further test. Otherwise, women underwent 100-g 3h-OGTT. GDM was diagnosed if two glucose values were above the thresholds including: FPG >95 mg/dl; 1-h glucose level >180 mg/dl; 2-h glucose level >155 mg/dl; and 3-h glucose level >140 mg/dl.

It should be noted that we excluded all those pregnant women who assigned to the protocol B in original study (25), since it was not a standard One-step approach and there was no corresponding group in the Two-step screening approach. Data on protocol B are presented in **Supplementary Tables 2**, **3**. In this protocol, GDM was defined as two or more of the given plasma glucose values are met or exceeded in One-step with 2-h 75 g oral glucose tolerance test.

**Figure 1:**
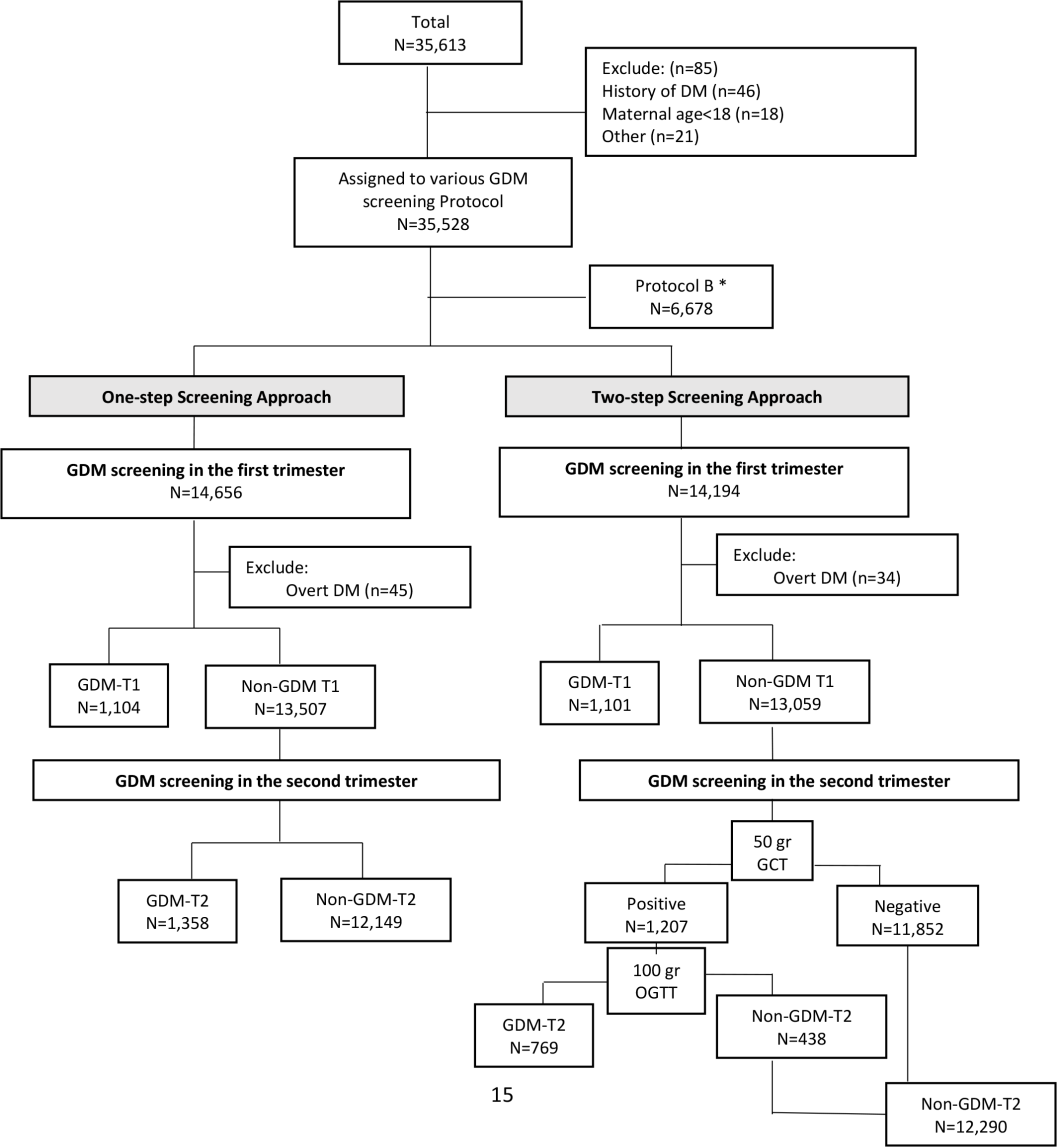
Flowchart of the study. *We excluded protocol B since it was not exact comparable with other standard approaches. In this protocol, GDM was defined as *two or more* of the given plasma glucose values are met or exceeded in One-step with 2-h 75 g oral glucose tolerance test. GDM: gestational Diabetes Mellitus; T1: first trimester of pregnancy; T2: second trimester of pregnancy.

All study participants were followed until delivery, and their outcomes were recorded in detail. Those pregnant women with a GDM diagnosis, either with a One- or Two-step approach, received specific prenatal and diabetic care, as recommended by the ACOG and the American Diabetes Association (2016) (26), including physical exercise, dietary intervention, and medication therapy (if necessary). The flowchart of the current study is presented in **Figure 1**.

### Outcomes and definition of terms

Macrosomia/large for gestational age (LGA) and primary cesarean section (C-S) were considered primary outcomes. Secondary outcomes were preterm birth before 37 weeks of gestation, admission to the neonatal intensive care unit (NICU), neonatal hypoglycemia, neonatal hypocalcemia, neonatal hyperbilirubinemia, preeclampsia, birth trauma, low birth weight (LBW), and intrauterine fetal death (IUFD).

Outcomes of the study were defined as follows: Macrosomia/large for gestational age (LGA) was defined as birth weight >4000 g and/or fetal weight >90th percentile for a given gestational age (27) using ultrasound biometry for estimating the fetal-weight and multinational World Health Organization (WHO) fetal growth chart for defining the percentile. Primary cesarean section was defined as the cesarean deliveries out of all births to women who had not had a previous cesarean. Hypoglycemia was defined as plasma glucose concentration <2.6 mmol/L in the first 48 h after delivery (28). Hyperbilirubinemia was determined by a value greater than the 95th percentile for any given point after birth; Preeclampsia was defined as an elevation in blood pressure ≥140 mmHg systolic or ≥90 mmHg diastolic on two occasions at least 4 h apart after 20 weeks of gestation in women with previously normal blood pressure and proteinuria ≥300 mg per 24 h urine collection or protein/creatinine ratio greater than or equal to 0.3 or dipstick reading of 1+ and more if other quantitative methods were not available; in the absence of proteinuria, new-onset hypertension with the new onset of any of the thrombocytopenia, renal insufficiency, impaired liver function, pulmonary edema, and cerebral or visual symptoms (29). Preterm birth was defined as when birth occurs between 20 and 37 weeks of pregnancy (30); Birth trauma was defined as brachial plexus palsy or clavicular, humeral, or skull fracture. LBW is defined as weight less than 2500 g at birth and/or fetal weight >90th percentile for a given gestational age using ultrasound biometry for estimating the fetal-weight and multinational World Health Organization (WHO) fetal growth chart for defining the percentile.

### Statistical analysis

Continuous variables were expressed as mean (standard deviation), and categorical variables were expressed as numbers (percentage). Characteristics of participants were compared between the two groups, by applying the independent *t*-test or Pearson’s chi-squared test for continuous and categorical data, respectively.

A modified Poisson regression for binary outcome data with a log link function and robust error variance was used to estimate relative risks (RRs) and 95% confidence intervals (CIs) for the associations between type of test and incidence of pregnancy outcomes. Adjusted variables were gestational ages at entrance and delivery, maternal BMI, gestational weight gain, type of delivery, and treatment modality. Moreover, we adjusted the GDM diagnosis in the model for comparing one or two abnormal tests. Both unadjusted and adjusted models were fitted. In all analyses related to the primary C-S outcome, those with a previous history of C-S were excluded. Penalized maximum likelihood estimation was applied in the case of sparse data.

Since the study was a cluster randomized trial, the cluster effect was considered in the analysis. Finally, the plot of the relative risk was depicted for all pregnancy outcomes by type of test. Statistical analysis was performed using STATA (version 13; STATA Inc., College Station, TX, USA), and the significance level of the test was set as 0.05.

## Results

Overall, 28,771 eligible pregnant women were assigned to One-step (n = 14611) or Two-step (n = 14160) screening for GDM. The characteristics of the women in the two groups are presented in **Table 1**. The mean maternal age and BMI of pregnant women in the One- and Two-step groups were [29.5 (5.9) vs. 29.5 (5.8) years] and [25.3 (4.8) vs. 25.9 (4.8) kg/m^2^], respectively. GDM was diagnosed in 9.3% of the pregnant women who were assigned to the One-step and in 5.4% of those assigned to the Two-step approach with a significant difference between them (p < 0.001).

**Table 1:** Characteristics of study participants.

Characteristics	One-Step Screening n = 14611	Two-Step Screening n = 14160
Time of screening, median (IQR), weeks		
First trimester,	8.6 (6.7–11)	8.7 (6.7–11.1)
Second trimester	26.1 (24.3-27.5)	26.3 (24.5-27.6)
Background characteristics		
Age, year	29.9 (5.8)	29.5 (5.9)
BMI at first trimester, kg/m^2^	25.9 (4.8)	25.3 (4.8)
<18.5, kg/m^2^, n (%)	475 (3.2)	692 (4.8)
18.5–24.9, kg/m^2^, n (%)	4350 (29.7)	4886 (34.51)
25.0–29.9, kg/m^2^, n (%)	4021 (27.5)	3837 (27.1)
≥30, kg/m^2^, n (%)	5765 (39.4)	4745 (33.5)
Gestational age at enrollment, weeks	9.2 (3.8)	9.2 (3.8)
Gestational age at delivery, weeks	38.7 (1.7)	38.7 (1.8)
Educational level, n (%)		
Elementary school	6 (0.04)	34 (0.24)
High school or diploma	9 (0.06)	42 (0.3)
College degree	4 (0.03)	23 (0.2)
Gravity	2.1 (1.1)	2.2 (1.2)
Parity	1.0 (0.9)	1.1 (0.9)
Parity ≥1, n (%)	7771 (53.2)	8206 (58.0)
Number of abortion	0.4 (0.7)	0.3 (0.6)
Systolic Blood Pressure	101.2 (9.4)	100.4 (9.8)
Diastolic Blood Pressure	63.5 (7.4)	62.8 (7.7)
Past history of adverse pregnancy outcomes*		
Gestational hypertension/preeclampsia, n (%)	202 (1.4)	216 (1.5)
Macrosomia, n (%)	135 (0.9)	206 (1.5)
Preterm birth, n (%)	245 (1.7)	237 (1.7)
Low Birth Weight, n (%)	371 (2.5)	362 (2.6)
GDM, n (%)	220 (1.5)	212 (1.5)
3^rd^ trimester vaginal bleeding, n (%)	41 (0.3)	45 (0.3)
Sever hemorrhage after delivery, n (%)	39 (0.3)	25 (0.2)
Fetal anomalies, n (%)	82 (0.6)	99 (0.7)
Twin pregnancy, n (%)	67 (0.5)	89 (0.6)
Still birth, n (%)	94 (0.6)	122 (0.9)
Instrumental delivery, n (%)	13 (0.1)	12 (0.1)
Family past medical history		
Type 2 diabetes Mellitus, n (%)	1318 (9.0)	1516 (10.7)
Chronic hypertension, n (%)	1629 (11.1)	1911 (13.5)
Protocol characteristic		
Protocol adherence, n (%)	13558 (92.7)	12511 (88.3)

Values are presented in Mean (SD) or Number (percentage) as appropriate;

BMI, Body mass index; GDM, gestational diabetes mellitus.

**Figure 2:**
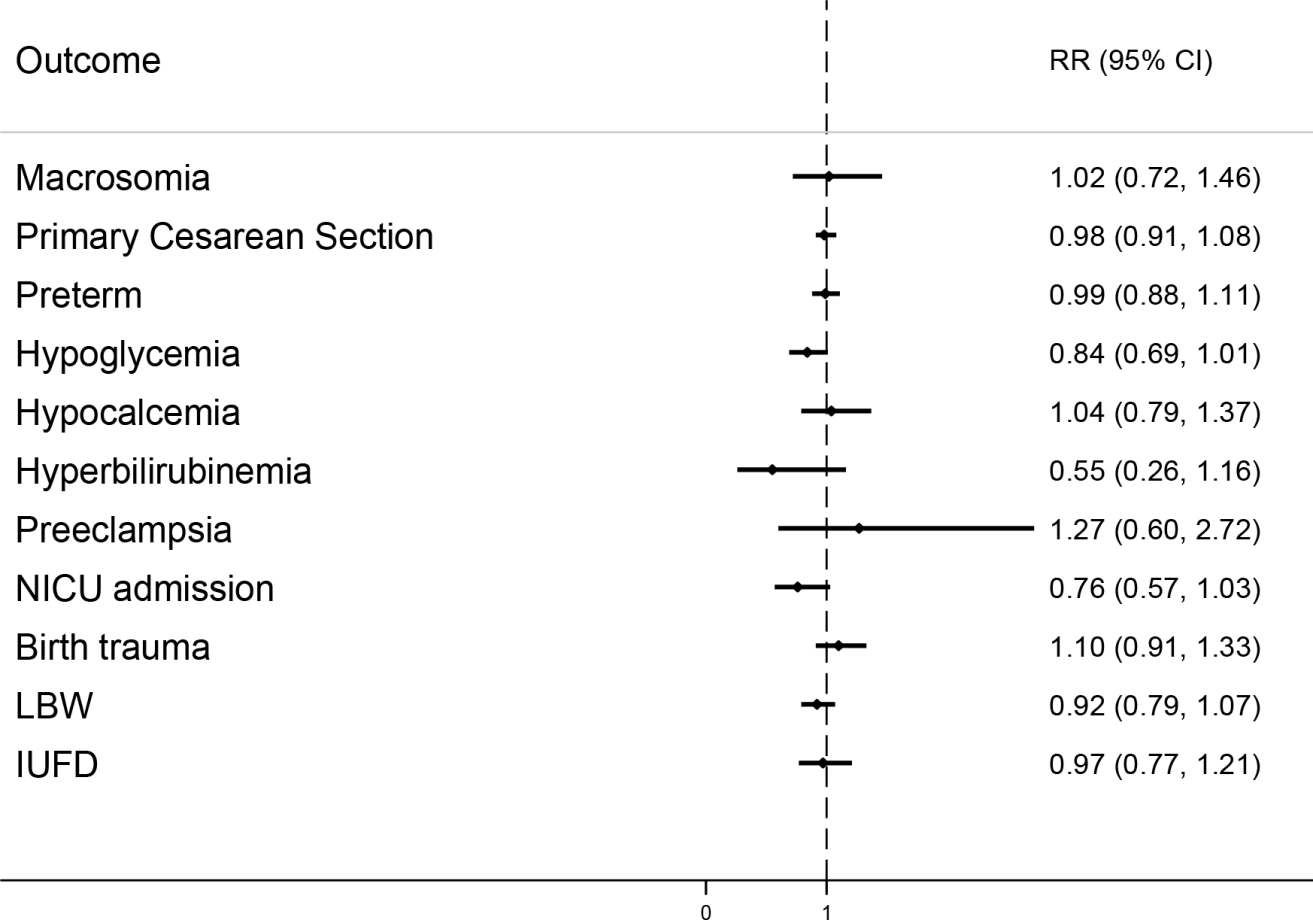
Adjusted risk ratio plot for pregnancy outcomes comparing Two-step vs. One-step.

The prevalence of maternal and neonatal outcomes in pregnant women based on the type of GDM screening approach and risk ratio (95% CI), comparing those outcomes in the Two-step versus One-step screening, is presented in **Table 2**. Intention-to-treat analyses showed no significant differences between the One-step and the Two-step group in the unadjusted risks the of the adverse pregnancy outcomes including primary outcomes of macrosomia (RR = 0.96; 95% CI: 0.64–1.45; P=0.821) and primary C-S (RR = 1.04; 95% CI: 0.88–1.23; p = 0.679), and also secondary outcomes of preterm birth, hypoglycemia, hypocalcemia, hyperbilirubinemia, preeclampsia, NICU admission, birth trauma, LBW, and IUFD. The results remained unchanged after adjustment for potential confounder variables including gestational age at enrollment and delivery, maternal BMI, gestational weight gain, type of delivery, treatment modality, and GDM diagnosis in the first trimester (**Table 2**; **Figure 2**). We re-analyzed these data after excluding those with diagnosis of GDM at first trimester. However, the findings remained unchanged (**Supplementary Table 4**).

**Table 2:** The prevalence of maternal and neonatal outcomes in pregnant women based on type of GDM screening approach and risk ratio (95% CI), comparing those outcomes in Two-Step versus One-Step screening.

	Prevalence		Risk Ratio			
Outcomes, n (%)	One-Step	Two-Step	Unadjusted		Adjusted	
	n = 14611	n = 14160	RR (95% CI)	p-Value	RR (95% CI)	p-Value
Macrosomia	835 (5.7)	779 (5.5)	0.96 (0.64–1.45)	0.8	1.02 (0.72–1.46)	0.9
Primary cesarean-section ^¥^	2232 (19.3)	2149 (20.2)	1.04 (0.88–1.23)	0.6	0.98 (0.91–1.08)	0.8
Preterm birth ^§^	852 (5.8)	882 (6.2)	1.06 (0.98–1.15)	0.1	0.99 (0.88–1.11)	0.8
Hypoglycemia	118 (0.8)	105 (0.7)	0.91 (0.74–1.13)	0.4	0.84 (0.69–1.01)	0.06
Hypocalcemia	78 (0.5)	80 (0.6)	1.05 (0.72–1.53)	0.8	1.04 (0.79–1.37)	0.8
Hyperbilirubinemia	1231 (8.5)	728 (5.1)	0.59 (0.26–1.35)	0.2	0.55 (0.26–1.16)	0.2
UV therapy	973 (79)	577 (79.2)	0.44 (0.18–1.87)	0.2	0.44 (0.18–1.73)	0.2
Preeclampsia	1312 (9.0)	1484 (10.5)	1.16 (0.56–2.39)	0.7	1.27 (0.60–2.72)	0.5
NICU admission	770 (5.3)	648 (4.6)	0.86 (0.64–1.17)	0.3	0.76 (0.57–1.03)	0.08
Birth trauma	65 (0.5)	84 (0.6)	1.33 (0.98–1.79)	0.07	1.10 (0.91–1.33)	0.3
Low Birth Weight ^€^	1228 (8.8)	1270 (9.4)	1.04 (0.89–1.21)	0.6	0.92 (0.79–1.07)	0.3
IUFD	87 (0.6)	103 (0.7)	1.20 (0.85–1.70)	0.3	0.97 (0.77–1.21)	0.8

*Adjusted variables were gestational age at enrollment and delivery, maternal BMI, gestational weight gain, type of delivery, treatment, and GDM diagnosis in the first trimester; ^¥^Outcome of primary cesarean-section women with repeated C-section were excluded, ^€^Outcome of LBW women with abortion were excluded, ^§^ For outcome of preterm birth, gestational age at delivery was not adjusted. RR, risk ratio; NICU, neonatal intensive care unit; IUFD, Intrauterine fetal death.

Reference group is One-step protocol.

## Discussion

In this secondary analysis of a randomized field trial among pregnant women, we found that the rates of GDM more than doubled with the One-step strategy. However, despite a significantly larger percentage of women who were diagnosed and subsequently managed for GDM in the One-step approach, the risk of adverse maternal and neonatal outcomes were similar in both groups, suggesting that many of the cases diagnosed with the lower threshold of the One-step method may not require specific GDM treatment.

Up to now, there is no worldwide consensus about optimum approach to diagnose GDM. Major scientific bodies acknowledge both One- and Two-step screening methods of GDM, but emphasize the need for additional evidence related to outcomes (1, 11–13, 26). Nonetheless, each approach has some advantages and disadvantages. In this respect, the One-step approach screens all pregnant women and screening and diagnosis could be completed in one visit; but all participants must be fast before screening and make time for a 2-h procedure. However, limiting the screening phase results in a single diagnostic test, which would be more convenient for the patient because it is better tolerated as it induces less nausea. It is more convenient for the provider; it could reduce the delay in diagnosis and care management (31). Besides, this approach could detect women with the milder form of hyperglycemia as having GDM, which potentially leads to a higher rate of GDM in the population, while the effects of identifying and treating milder cases of GDM on adverse pregnancy outcomes are not clearly understood (1, 32). It is also uncertain whether this increase in the diagnosis of GDM will affect the behavior and intention of physicians as to cesarean delivery, NICU admission, or induction of labor (20). As such, the Two-step approach is simpler for individuals, since it does not need to be fast and can easily be done as part of a scheduled prenatal visit. In addition, approximately 80% of pregnant women do not require further next step screening (33, 34).

The results of our study are in agreement with the majority of the available literature that the One-step approach led to higher GDM prevalence among pregnant women (15, 35). There were a limited number of studies that reported better pregnancy outcomes by applying the One-step approach for the screening and diagnosis of GDM. In this respect, there is no consensus between major scientific bodies regarding the One- or Two-step approach recommendations for GDM screening in the second trimester of gestation.

In agreement with our findings, in recently published meta-analyses, which involved 50 population-based studies and more than 1.5 million pregnant women with GDM and 7.5 million non-GDM counterparts, it was reported that applying the IADPSG criteria for GDM diagnosis could significantly increase the prevalence of GDM, while it could not improve the risk of adverse pregnancy outcome compared other criteria (2, 3). However, it should be noted that most of the included studies in those meta-analyses were observational and therefore the findings may be vulnerable to systematic errors such as bias and confounding (36). Nevertheless, a limited number of five RCTs have been published so far, which have conflicting results (15, 17, 20, 22, 37). In the largest trial, Hillier et al. (15) compared the One- and Two-step approaches of screening and diagnosis of GDM among 23,792 eligible pregnant women. In agreement of our findings, they demonstrated that despite a doubling in the incidence of GDM diagnosis with the One-step approach, there were no significant between-group differences in the risks of adverse outcomes of LGA infants (8.9% vs. 9.2%), gestational hypertension or preeclampsia (13.6% vs. 13.5%), primary cesarean delivery (24% vs. 24.6%), or the primary composite outcome including stillbirth, neonatal death, shoulder dystocia, bone fracture, and/or any upper extremity nerve palsy related to birth injury (3.1% vs. 3.0%) (15). Likewise, Satodiya et al. (19) performed a prospective RCT, involving 1000 pregnant women, to compare the incidence and the maternal and fetal outcomes of GDM using the One-step versus Two-step method. They found that the incidence of GDM using the One-step was almost double versus the Two-step approach, but the maternal and fetal outcomes were comparable (19). In contrast, Sevket et al. (20), in another randomized trial, compared the prevalence and clinical outcomes of a One-step (n = 386) with a Two-step screening (n = 400) method. It is reported that women who were defined as having normal glucose tolerance by the One-step screening method had better perinatal outcomes than women who were defined as having normal glucose tolerance by the Two-step screening method (20). Difference in setting and population characteristics and also adjustment for different potential confounders may potentially lead to this inconsistency in the results. In an RCT meta-analysis of three studies, it is shown that the prevalence of GDM in both One- and Two-step approaches were similar (8.4% vs. 4.3%; relative risk 1.64, 95% CI, 0.77–3.48 and the One-step approach was associated with better maternal and perinatal outcomes (18). However, the results of this RCT meta-analysis should always be interpreted with caution as only three RCTs were included in this meta-analysis. The largest one had not reported data on maternal and perinatal outcomes.

However, the findings of our community randomized trial showed that while the adoption of One-step screening could increase the prevalence of GDM, it could, however, not improve pregnancy outcomes. The results of this study are clinically valuable because this is the first community-based trial in a developing country where the rate of GDM is high (38) and the health resources are limited; therefore, it is important to balance the risks of increasing the diagnosis of GDM, such as increased healthcare utilization and cost (39).

Additionally, the over-diagnosis may lead to psychological presser and impaired quality of life of pregnant women (40). Individual with GDM and their families are challenged with complex, multifaceted, and emotional issues when integrating specific care into daily life. Along with concerns about fetal health, this not only places an emotional burden, but may also invite negative attention, comment, and judgment from others, suggesting a socioemotional burden (41).

It should be noted that the rate of macrosomia in this study was low. We hypothesized that this phenomenon may be related to ethnicity differences of Iranian population (42) and also strict management and monitoring of GDM cases in the current study.

However, this study was one of the largest community-based trials in the developing country that may cover the gap of knowledge that arises from previous studies. One of the important strengths of our study is the generalizability of findings due to its characteristics, including its design as a community-based trial, large sample size, the geographic distribution of the regions involved, and using similar laboratory protocols. In addition, the adherence to each protocol in our study was high (25), which could increase the validity of our findings.

Nevertheless, we were limited by excluding women with known chronic disorders, since based on our national guidelines, those women should visit the second and third level of the health care delivery system directly and not receive prenatal care in a primary healthcare setting, which was the platform of our study. Moreover, in this study, we did not assess the long-term benefits of the One- and Two-step approaches of GDM screening, such as improved long-term metabolic or cardiovascular health, for mothers and their offspring. We did not have the results of IV therapy for those neonates with hypoglycemia. Additionally, a central reference laboratory was not used for all our measurements, though all laboratory procedures, equipment, and supplies were homogeneous in different geographical regions of the study, and monthly external quality controls were performed for each laboratory to confirm the validity and reliability of all laboratory measures.

## Conclusions

In this large randomized community-based trial of a population of pregnant women who undergoing GDM screening by either the One-step or Two-step approach, we found that despite a significantly larger rate of women who were diagnosed and subsequently managed for GDM in One-step approach, two approaches were similar in terms of maternal and neonatal outcomes. These findings may warn that more caution should be exercised in adopting the One-step method worldwide. Future research is needed to assess the long term harm and benefits of those approaches of GDM screening for both mothers and their offspring.

## Data availability statement

The raw data supporting the conclusions of this article will be made available by the authors, without undue reservation.

## Ethics statement

This trial has been approved and funded by the National Institute for Medical Research Development under Grant Agreement No IR.NIMAD.REC.1394.013. Funding source had no involvement in the study. The protocol was approved by the national ethics committee of the National Institute for Medical Research Development (Approval number: IR.NIMAD.REC.1394.013). In addition, the Iranian Ministry of Health and Medical Education (MoHME) approved the study protocol and pre specified GDM modalities were made available to all those provinces as mandatory guidelines. This field trial has been registered in Iranian Registry of Clinical Trials (Trial Registration: IRCT138707081281N1). Written informed consent for participation was not required for this study in accordance with the national legislation and the institutional requirements.

## Author contributions

FRT and MR had full access to all of the data in the study and take responsibility for the integrity of the data and the accuracy of the data analysis. Concept and design: FRT, SB-G, FF, MA, FHo, FHa, FT, FA. Acquisition, analysis, or interpretation of data: FRT, MR, SB-G, FHo. Drafting of the manuscript: FRT, MR, SB-G. Critical revision of the manuscript for important intellectual content: FF, MA, MF, FHo, FHa, MV, FA. Supervision: FRT, FA, SB-G. All authors contributed to the article and approved the submitted version.
