# Supplementary material for: One-step versus two-step screening for diagnosis of gestational diabetes mellitus in Iranian population: A randomized community trial
Source: Front Endocrinol (Lausanne). 2023 Feb 2;13:1039643. doi: 10.3389/fendo.2022.1039643 (PMC9932332; doi:10.3389/fendo.2022.1039643)
Supplement: Supplementary file 1 [file DataSheet_1.docx]

All provinces in Iran

North of Iran (**Golestan province)**

West of Iran (**Kurdistan province)**

Center of Iran (**Yazd province)**

East of Iran (**South Khorasan province)**

South of Iran (**Bushehr province)**

Stratification according to the geographical region

Clustering based on socio-economic situation

First cluster: centers of provinces

Second cluster: Other cities in each province

*Random allocation* of protocol among center of provinces

**Golestan province**: Gorgan city (E)

**Kurdistan province**: Sanandaj city (B)

**Yazd province**: Yazd city (C)

**South Khorasan province**: Birjand city (D)

**Bushehr province**: Bushehr city (A)

*Random allocation* of protocol among 4 cities in each province

**Golestan Province**

City 1: Gonbad (C)

City 2: Agh-ghela (D)

City 3: Torkaman (A)

City 4: Ali-abad (B)

**Kurdistan Province**

City 1: Saghez (A)

City 2: Ghorveh (E)

City 3: Marivan (C)

City 4: Baneh (D)

**Yazd Province**

City 1: Ardakan (D)

City 2: Mehriz (B)

City 3: Meibod (E)

City 4: Bafgh (A)

**Bushehr Province**

City 1: Dashtestan (C)

City 2: Dashti (D)

City 3: Gonaveh (E)

City 4: Kangan (B)

**South Khorasan Province**

City 1: Ghaen (E)

City 2: Ferdous (C)

City 3: Tabas (A)

City 4: Nehbandan (B)

Random selection of 4 cities in each provinces

Supplementary Figure 1: Randomization process of study

Supplementary Table 1. Baseline characteristics for participants in protocol B.

| Characteristics | | Protocol B  n = 6,659 |
| --- | --- | --- |
| Background characteristics | |  |
| Age, year | | 30.6(5.8) |
| BMI at first trimester, kg/m^2^ | | 26.5(4.7) |
| Gestational age at enrollment, week | | 8.6(3.3) |
| Gestational age at delivery, week | | 38.9(1.8) |
| Educational level, n(%) | |  |
|  | Elementary School | 197(3) |
|  | High school or Diploma | 462(7) |
|  | College degree | 179(2.7) |
| Gravity | | 2.1(1.1) |
| Parity | | 1.1(0.9) |
|  | Parity ≥1, n(%) | 3239(48.6) |
| Number of abortion | | 0.5(0.8) |
| Systolic Blood Pressure | | 102.4(9.4) |
| Diastolic Blood Pressure | | 64.9(6.8) |
| Past history of adverse pregnancy outcomes | |  |
| Gestational hypertension / preeclampsia, n(%) | | 84(1.3) |
| Macrosomia, n(%) | | 64(1.0) |
| Preterm birth, n(%) | | 122(1.8) |
| Low Birth Weight, n(%) | | 153(2.3) |
| GDM, n(%) | | 101(1.5) |
| 3^rd^ trimester vaginal bleeding, n(%) | | 12(0.2) |
| Sever hemorrhage after delivery, n(%) | | 10(0.15) |
| Fetal anomalies, n(%) | | 29(0.44) |
| Twin pregnancy, n(%) | | 44(0.66) |
| Still birth, n(%) | | 59(0.9) |
| Instrumental delivery, n(%) | | 13(0.2) |
| Family past medical history | |  |
| Type 2 diabetes Mellitus, n(%) | | 655(9.8) |
| Chronic hypertension, n(%) | | 977(14.7) |

Supplementary Table 2. Prevalence of adverse pregnancy outcomes in participants based on protocol B.

| adverse pregnancy outcomes | Protocol B  n = 6,659 |
| --- | --- |
| Macrosomia | 373(5.6) |
| Primary cesarean-section | 882(15.8) |
| Preterm birth | 395(5.9) |
| Hypoglycemia | 61(0.9) |
| Hypocalcemia | 34(0.5) |
| Hyperbilirubinemia | 440(6.6) |
| Preeclampsia | 793(11.9) |
| NICU admission | 297(4.5) |
| Birth trauma | 48(0.7) |
| Low Birth Weight | 509(8.1) |
| IUFD | 52(0.8) |

Supplementary table 3. results of pharmacotherapy


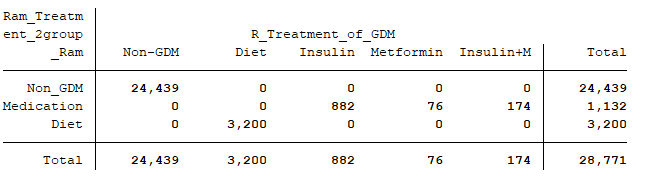


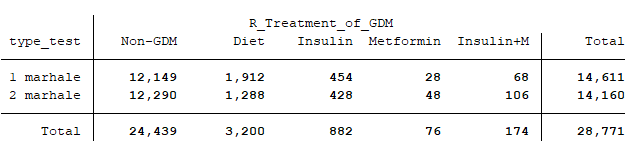


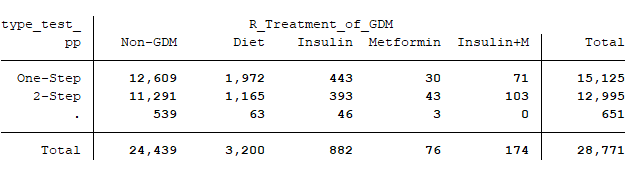


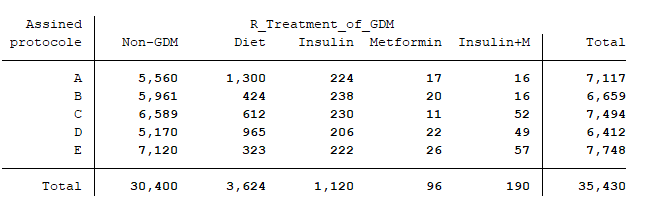


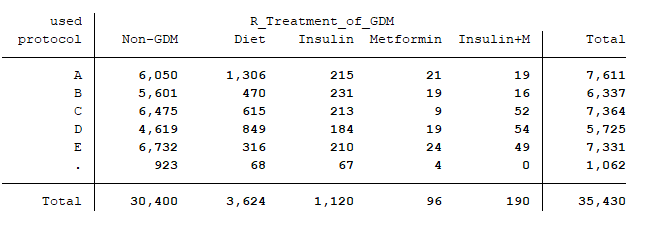


Supplementary Table 4. The prevalence of maternal and neonatal outcomes in pregnant women based on type of GDM screening approach and risk ratio (95% CI), comparing those outcomes in Two-Step versus One-Step screening (after excluding those with diagnosis of GDM at first trimester).

|  | Prevalence | | Risk Ratio | | | |
| --- | --- | --- | --- | --- | --- | --- |
| Outcomes | One-Step | Two-Step | Unadjusted | | Adjusted | |
|  | n = 13507 | n = 13059 | RR (95% CI) | P-value | RR (95% CI) | P-value |
| Macrosomia | 740 (5.5) | 691 (5.3) | 0.97 (0.63-1.49) | 0.9 | 1.01 (0.70-1.46) | 0.9 |
| Primary cesarean-section ^¥^ | 2065 (19.3) | 1997 (20.3) | 1.04 (0.88-1.23) | 0.6 | 1.00 (0.92-1.08) | 0.9 |
| Preterm birth ^§^ | 780 (5.8) | 782 (6.0) | 1.03 (0.92-1.15) | 0.6 | 0.97 (0.83-1.13) | 0.7 |
| Hypoglycemia | 93 (0.7) | 66 (0.5) | 0.73 (0.61-0.87) | **0.001** | 0.81 (0.53-1.22) | 0.3 |
| Hypocalcemia | 58 (0.4) | 49 (0.4) | 0.87 (0.50-1.55) | 0.6 | 1.00 (0.60-1.68) | 0.9 |
| Hyperbilirubinemia | 1133 (8.4) | 673 (5.1) | 0.60 (0.27-1.33) | 0.2 | 0.56 (0.27-1.16) | 0.1 |
| Preeclampsia | 1208 (8.9) | 1335 (10.2) | 1.14 (0.55-2.35) | 0.7 | 1.26 (0.59-2.71) | 0.5 |
| NICU admission | 690 (5.1) | 563 (4.3) | 0.84 (0.64-1.09) | 0.2 | 0.75 (0.58-0.98) | **0.03** |
| Birth trauma | 59 (0.4) | 75 (0.6) | 1.31 (0.98-1.73) | 0.06 | 1.14 (0.92-1.42) | 0.2 |
| Low Birth Weight ^€^ | 1147 (8.9) | 1162 (9.3) | 1.02 (0.88-1.18) | 0.8 | 0.92 (0.79-1.07) | 0.3 |
| IUFD | 76 (0.6) | 92 (0.7) | 1.23 (0.87-1.75) | 0.2 | 1.04 (0.81-1.33) | 0.8 |
| * Adjusted variables were gestational age at enrollment and delivery, maternal BMI, gestational weight gain, type of delivery, treatment, and GDM diagnosis in the first trimester; ^¥^ For outcome of primary cesarean-section women with repeated C-section were excluded, ^€^ For outcome of LBW women with abortion were excluded, ^§^ For outcome of preterm birth, gestational age at delivery was not adjusted. Bold values indicate statistical significance at level 0.05; RR: risk ratio, NICU: neonatal intensive care unit; IUFD: Intrauterine fetal death.  Reference group is one step protocol. | | | | | | |
